# Niacin Inhibits Vascular Inflammation via Downregulating Nuclear Transcription Factor-**κ**B Signaling Pathway

**DOI:** 10.1155/2014/263786

**Published:** 2014-05-27

**Authors:** Yanhong Si, Ying Zhang, Jilong Zhao, Shoudong Guo, Lei Zhai, Shutong Yao, Hui Sang, Nana Yang, Guohua Song, Jue Gu, Shucun Qin

**Affiliations:** ^1^Key Laboratory of Atherosclerosis in Universities of Shandong and Institute of Atherosclerosis, Taishan Medical University, Shandong 271000, China; ^2^College of Basic Medical Sciences, Taishan Medical University, Shandong 271000, China; ^3^Shandong Agricultural University, Shandong 271000, China; ^4^Department of Geriatrics, PLA Navy General Hospital, Beijing 100048, China

## Abstract

The study aimed to investigate the effect of niacin on vascular inflammatory lesions *in vivo* and *in vitro* as well as its lipid-regulating mechanism. *In vivo* study revealed that niacin downregulated the levels of inflammatory factors (IL-6 and TNF-**α**) in plasma, suppressed protein expression of CD68 and NF-**κ**B p65 in arterial wall, and attenuated oxidative stress in guinea pigs that have been fed high fat diet. *In vitro* study further confirmed that niacin decreased the secretion of IL-6 and TNF-**α** and inhibited NF-**κ**B p65 and notch1 protein expression in oxLDL-stimulated HUVECs and THP-1 macrophages. Moreover, niacin attenuated oxLDL-induced apoptosis of HUVECs as well. In addition, niacin significantly lessened lipid deposition in arterial wall, increased HDL-C and apoA levels and decreased TG and non-HDL-C levels in plasma, and upregulated the mRNA amount of cholesterol 7**α**-hydroxylase A1 in liver of guinea pigs. These data suggest for the first time that niacin inhibits vascular inflammation *in vivo* and *in vitro* via downregulating NF-**κ**B signaling pathway. Furthermore, niacin also modulates plasma lipid by upregulating the expression of factors involved in the process of reverse cholesterol transport.

## 1. Introduction

Niacin (nicotinic acid, vitamin B3) is a water-soluble vitamin. In 1955, Altschul et al. reported for the first time that pharmacological doses of niacin can lower plasma cholesterol level in normal persons as well as hypercholesterolemic patients [1]. Several subsequent clinical studies established the usage of niacin as a broad-spectrum lipid-regulating medication. Niacin alone or in combination can slow or reverse the progression of atherosclerosis (AS) and reduce cardiovascular event rates and total mortality in patients with hypercholesterolemia and atherosclerotic cardiovascular disease [2, 3]. These effects are generally attributed to favorable actions on the lipoprotein profile, which includes LDL-C reduction and HDL-C elevation. However, it is not clear whether the beneficial effects of niacin on cardiovascular disease can be completely explained by alterations of plasma lipids.

Recent studies indicated niacin also has the anti-inflammatory and antioxidant properties. In human aortic endothelial cells* in vitro*, niacin significantly suppressed the adhesion and accumulation of monocytes/macrophages and inhibited angiotensin II-induced reactive oxygen species (ROS) production and LDL oxidation [4], but it is unknown whether niacin has the same effects or not* in vivo*. In addition, Kuvin et al. confirmed that extended-release niaspan not only had a beneficial lipid-regulating effect but also decreased C-reactive protein (CRP) by 15% in patients with stable coronary artery disease [5]. CRP is one of the most important inflammatory markers. Therefore, we hypothesize that niacin has vascular anti-inflammatory and potentially vascular-protective property independent of its effect on lipid regulation.

Guinea pigs have been successfully used to study the damage of hyperlipidemia [6]. Like human, guinea pig is one of the few species that carry the majority of cholesterol in LDL [7]. It is possible to induce the initial stages of AS quickly by feeding them high fat diet [8]. In addition, significant atherogenic inflammation with an increase of aortic cytokines has been documented in guinea pigs [7]. In the study, we select guinea pigs as animal model to study the protective effect of niacin on vascular inflammatory lesions induced by high fat diet* in vivo*. Meanwhile, to further confirm the direct anti-inflammatory property of niacin, its effect on oxLDL-induced inflammation of endothelial cells and macrophages is also explored.

## 2. Materials and Methods

### 2.1. Animal and Experimental Design

Thirty-two male England short-hair guinea pigs (260~310 g; 5 months old) were bought from the Animal Laboratory Center of Taibang Biological Products Co., Shandong, China. All experiments were approved by the Laboratory Animals' Ethical Committee of Taishan Medical University and abided by national guidelines for the care and use of animals. All guinea pigs were randomly divided into 4 groups: regular chow diet group (CD), high fat diet group (HFD, 10% lard + 10% yolk power + 0.30% cholesterol + 79.7% grass), HFD with niacin group (HFD-N,  HFD + 100 mg/kg/d niacin),  and  HFD  with  simvastatin  group  (HFD-S, HFD + 20 mg/kg/d simvastatin). Each group is assigned 8 guinea pigs. The drugs (niacin or simvastatin) were fed by oral gavage once daily for 8 weeks.

### 2.2. Cell Culture

Human umbilical vein endothelial cells (HUVECs, EA.hy926) and THP-1 (human monocyte) were all purchased from Shanghai Institute of Biochemistry. They were maintained in RPMI 1640 medium (HyClone, China) supplemented with 10% fetal bovine serum (HyClone, China) in a 5% CO2 incubator at 37°C. To induce monocytes differentiation into macrophages, THP-1 cells were cultured with 50 ng/mL phorbol 12-myristate 13-acetate (PMA; Sigma) for 24 hours, as described previously [9].

### 2.3. Immunohistochemistry Examination

Tissue sections (5 *μ*m) from formalin-fixed, paraffin-embedded specimens were stained with specific antibodies against NF-*κ*B p65 and CD68 proteins (Zhongshan Biotechnology Co., Ltd., Beijing, China), respectively. The sections were developed with 3,3′-diaminobenzidine (Vector Laboratories) and counterstained with Mayer's hematoxylin (Saturatedard Allen). Images were captured using microscope (Olympus). All quantifications were examined by calculating the percentage of integrate optical density (IOD) of the antigen positive staining to the entire cross-sectional vessel wall by Image-Pro Plus software.

### 2.4. Analysis of Lipid Deposition in the Arterial Wall

The proximal aorta attached to the heart was applied to prepare cryosections. Cryosections (8 *μ*m) were cut, gathered, and stained with oil red O. The quantification of stained lipids was examined by calculating the percentage of the positive area to the total cross-sectional vessel wall area by using Image-Pro Plus software 4.5 (Media Cybernetics). The percentage was calculated from five sections from an animal.

### 2.5. Measurement of Inflammatory Factors

Plasma concentrations of IL-6, TNF-*α*, and CRP, which can be secreted during the process of inflammation, were determined by ELISA kit (American R&D) in accordance with the manufacturer's instructions.

ELISA kits were also used for the measurement of TNF-*α* and IL-6 protein levels in the medium of HUVECs and THP-1 macrophages. Cells were incubated in the absence or presence of niacin (0.25–1 mM) for 24 h in serum-free media and then stimulated by 150 *μ*g/mL ox-LDL (Beijing Xiesheng Biotechnology Co. Ltd., China) for 6 h. TNF-*α* and IL-6 protein levels were assessed, respectively. Results were calculated as ng/L and expressed as a percentage of those obtained with blanks.

### 2.6. Measurement of Oxidative Stress Reaction in Plasma

Plasma level of malondialdehyde (MDA), a marker for oxidative stress, was determined by a spectrophotometric measurement of thiobarbituric acid-reactive substances (TBARS) in accordance with the manufacturer's instructions (Nanjing Jiancheng Bioengineering Institute, China).

### 2.7. Plasma Lipid Analysis

After being treated by niacin or simvastatin for 8 weeks, blood was collected by cardiac puncture from guinea pigs without dietary exposure for 12 hours. Concentrations of plasma total cholesterol (TC), triglyceride (TG), and HDL cholesterol (HDL-C) were determined by enzymatic methods (BioSino, Beijing, China). Non-HDL-C was calculated as TC minus HDL-C.

### 2.8. Sodium Dodecyl Sulfate Polyacrylamide Gel Electrophoresis (SDS-PAGE) Analysis of Apolipoproteins in HDL

Lipoprotein isolation was carried out by sequential ultracentrifugation in a LE-80 K ultracentrifuge (Beckman Coulter, Inc. Brea, CA, USA) as described before. Separation was done according to the following density fractionation:* d* < 1.019 g/mL for VLDL and IDL;* d* 1.019–1.09 g/mL for LDL; and* d* 1.09–1.24 g/mL for HDL [10]. The isolated specimens were dialyzed in 150 mmol/L NaCl and 0.3 mmol/L EDTA at 4°C. HDL containing equal amounts of cholesterol was loaded on a 15% sodium dodecyl sulfate (SDS) polyacrylamide gradient gel and apolipoprotein samples were stained with coomassie brilliant blue as described by Jiang et al. [11]. Meanwhile, the marker (Invitrogen, LC5800) was put in lane 1 and HDL from human was put in the last lane for contrast. Stained gels were scanned and analyzed by Quantity One (Bio-Rad, Hercules, CA, USA) software program.

### 2.9. Analysis of Cell Apoptosis by Flow Cytometry

Annexin V-FITC/PI double-staining assay was applied to measure apoptosis according to the manufacturer's instructions. After HUVECs were stimulated by ox-LDL for 24 h, cells were centrifuged, washed twice with PBS, resuspended in 500 uL binding buffer, and incubated with 5 uL fluorescein isothiocyanate (FITC)-labeled Annexin V and 5 uL propidium iodide (PI) for 10 minutes at room temperature in the dark. The scatter parameters of cells were analyzed by FAC Scan flow cytometer and Cell Quest analysis software (Becton-Dickinson, CA, USA). Four cell populations were discerned according to the following status: live cells in the lower-left quadrant (low-PI and FITC signals), early apoptotic cells in the lower-right quadrant (low-PI and high-FITC signals), late apoptotic or necrotic cells in the upper-right quadrant (high-PI and high-FITC signals), and necrotic cells in the upper-left quadrant (high-PI and low-FITC signals).

### 2.10. Western Blot

The entire proteins from fresh aortic walls or treated cells were extracted using RIPA lysis buffer. Then the nuclear protein fraction was prepared by a nuclear protein extraction kit (BestBio, China) in accordance with the manufacturer's instructions. Equal amounts of protein were subjected to 8% to 15% SDS-PAGE and transferred onto PVDF membranes by electroblotting. After blocking in Tris-buffered saline (TBS) containing 0.1% Tween 20 and 10% nonfat dry milk for 2 h at room temperature, the membranes were incubated with primary antibodies for 3 h at room temperature or overnight at 4°C. After being washed four times with TBS containing 0.1% Tween 20, the membranes were incubated with horseradish peroxidase-conjugated secondary antibodies for 1 h at room temperature. Immunoblots were revealed by ECL reaction and visualized using a high-performance chemiluminescence film. The IOD value of immunoreactive bands was measured by Image-Pro Plus software and normalized by house-keeping protein (*β*-actin or histone H3).

### 2.11. Quantitative Real-Time PCR

RT-PCR assay was applied to detect the expression of LDL receptor (LDL-R), scavenger receptor Class B Type 1 (SR-B1), 3-hydroxy-3-methyl-glutaryl-CoA reductase (HMGCR), and cholesterol 7*α*-hydroxylase A1 (CYP7A1) in liver. The first two are receptors of plasma cholesterol and the rest are key enzymes of cholesterol metabolism. Primer Design 4.1 Software was used to design the following primers:  *β*-actin:  forward  primer:  5′-TTACTACTTTGCTGCGTTACACC-3′, reverse primer: 5′-CATGCCAATCTCATCTCGTTT-3′ (length of 78 bp);  LDL-R:  forward  primer:  5′-GACGTGTCCCAGAGGAAGAT-3′,  reverse  primer:  5′-CGAGTCGGTCCAGTAGATGTT-3′ (length  of  144 bp);  SR-B1:  forward  primer:  5′-TCTCCCACCCGCATTTCT-3′,  reverse  primer:  5′-CGCATACTGCACGTAGCACA-3′ (length  of  317 bp); CYP7A1: forward primer: 5′-CAGTATGCTGCTGTTTATG-3′, reverse primer: 5′-GTTCTCGGTGGTGTTTCC-3′ (length  of  335 bp);  HMGR:  forward  primer:  5′-TGATAGCACCAGCAGATT-3′, reverse primer: 5′-TATAAAGGTTGCGTCCAG-3′ (length of 68 bp). The primers were synthesized by TaKaRa. Total liver RNA was separated by TRIZOL Reagent (Invitrogen). cDNA synthesis was performed using MuLV Reverse Transcriptase (Applied Biosystems). Real-time PCR was performed using a SYBR-green PCR master mix kit (TianGen Biotech). The data was analyzed by using Rotor-gene Q software ver.1.7 (Qiagen). Relative mRNA levels were calculated by the 2^−ΔΔCt^ method and normalized against *β*-actin. Each experiment was repeated three times.

### 2.12. Statistical Analysis

Results are shown as the mean ± SD for at least three independent experiments. Statistical analysis was performed using one-way analysis of variance (ANOVA) followed by Student-Newmann-Keuls multiple comparison tests with the SPSS 13.0 software for Windows. *P* values less than 0.05 were considered statistically significant.

## 3. Results

At the beginning of the experiment, 32 guinea pigs were divided into 4 groups randomly and mean initial body weight was 302.27 ± 23.67 g. All guinea pigs survived for 8 weeks in the experiment and mean final body weight was 384.89 ± 26.18 g. No significant differences were observed among these groups for both the initial and final mean body weights.

### 3.1. Niacin Attenuated the Systemic and Aortic Inflammation in Guinea Pigs Fed High Fat Diet

#### 3.1.1. Niacin Significantly Downregulated IL-6 and TNF-*α* Levels in Plasma of Guinea Pigs Fed High Fat Diet

Inflammatory process within the vessel wall can lead to vascular dysfunction and cause cardiovascular disease. In this process, inflammatory factors play a key role. In this study, the levels of three major inflammatory factors (CRP, IL-6, and TNF-*α*) in plasma were measured. Compared with CD group, high fat diet for 8 weeks lightly increased the levels of CRP, IL-6, and TNF-*α* in plasma, but the increase was not statistically significant (*P* > 0.05). Compared with HFD group, niacin decreased IL-6 level by 19% and decreased TNF-*α* level by 18%, whereas its effect on CRP had no statistical difference. Simvastatin decreased the levels of three inflammatory markers in plasma compared with HFD group (Table 1).

#### 3.1.2. Niacin Suppressed Protein Expression of CD68 and NF-*κ*B p65 in the Arterial Wall of Guinea Pigs Fed High Fat Diet

**Figure 1:**
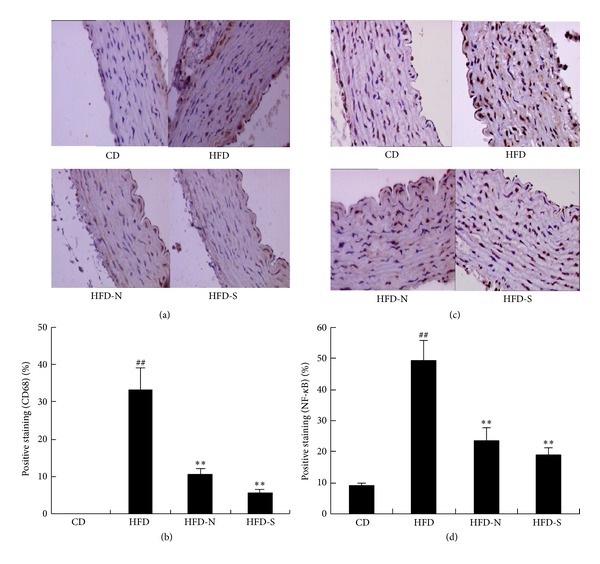
Niacin and simvastatin reduced the expressions of CD68 and NF-*κ*B p65 in the arterial wall of guinea pigs by immunohistochemistry analysis after treatment for 8 weeks. (a) and (c) show the representative immunostained aortic sections of CD68 and NF-*κ*B p65, respectively (20x magnification; blue = nuclei and brown = target protein). (b) and (d) show the relative levels of CD68 and NF-*κ*B positive cells of per view field by densitometric quantitation, respectively. Data are presented as mean ± SD (*n* = 8). ^##^ *P* < 0.01 versus CD group; **P < 0.01 versus HFD group.

The invasion of monocyte into arterial intima and its differentiation into resident macrophages may contribute to arterial inflammation in experimental atherosclerosis and hypertension. In the study, the immunohistochemical examination of CD68 protein in the vessel wall was done to mark monocytes/macrophages. Densitometric quantitative immunohistochemistry imaging revealed that, compared with CD group, the positive staining of CD68 was significantly increased in HFD group (*P* < 0.01); compared with HFD group, niacin and simvastatin significantly decreased the densitometric value of positive staining (Figures 1(a) and 1(b)). These results indicated that niacin weakened the adhesion and invasion of monocytes in the arterial wall.

**Figure 2:**
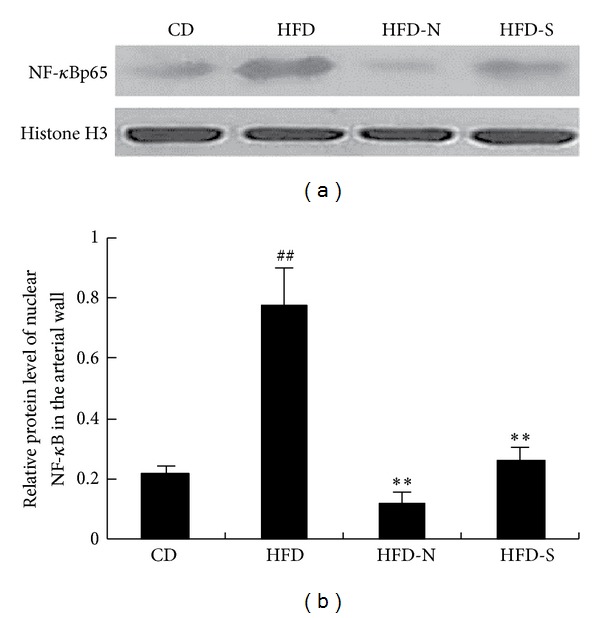
Niacin and simvastatin suppressed the expression of nuclear protein NF-*κ*B p65 in the arterial wall of guinea pigs fed high fat diet. The protein expression was analyzed by western blot and normalized to histone H3 level. (a) shows the representative image by western blot. (b) shows the IOD ratio of NF-*κ*B p65 to Histone H3. Data are presented as mean ± SD of at least three independent experiments. ^##^ *P* < 0.01 versus CD group; **P < 0.01 versus HFD group.

NF-*κ*B is a transcription factor associated with the expression of various proinflammatory mediators [12]. When not stimulated, it is found in the cytoplasm connected to its inhibiting protein, inhibitor *κ*B kinase (I*κ*B). Stimulation by inflammatory factors causes degradation of I*κ*B protein and then translocates NF-*κ*B to the nucleus. In nucleus, NF-*κ*B upregulates gene expressions of inflammatory molecules including chemokines, cytokines, adhesion molecules, and proteases [13]. In order to further explore the mechanism through which niacin inhibited inflammatory progress, we determined the expression of nuclear protein NF-*κ*B p65 in the arterial wall by immunohistochemistry analysis and western blot. The results all indicated that, compared with CD group, high fat diet promoted the expression of active NF-*κ*B p65 in the arterial wall (*P* < 0.01); compared with HFD group, niacin and simvastatin significantly decreased the expression (Figures 1(c), 1(d), 2(a), and 2(b)).

#### 3.1.3. Niacin Attenuated Oxidative Stress in Guinea Pigs Fed High Fat Diet

**Figure 3:**
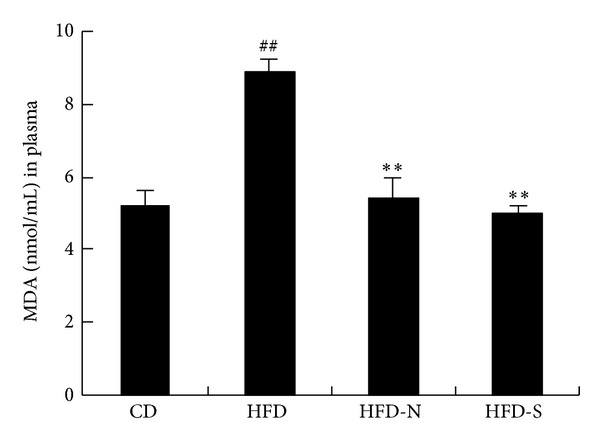
Niacin and simvastatin decreased the level of plasma MDA in guinea pigs after treatment for 8 weeks. MDA was determined by a spectrophotometric measurement of thiobarbituric acid-reactive substances (TBARS) according to the manufacturer's instruction. Data are presented as mean ± SD (*n* = 8). ^##^ *P* < 0.01 versus CD group; **P < 0.01 versus HFD  group.

Oxidative stress plays an important role in the inflammatory process [14]. MDA is one of the most reliable and widely used indices of oxidative stress [15]. In our study, we determined MDA level in plasma. As shown in Figure 7, compared with that of CD group, the level of MDA in plasma was significantly increased in HFD group (*P* < 0.01). Compared with that of HFD group, niacin and simvastatin significantly lowered the MDA level by 38% and 43%, respectively (Figure 3).

### 3.2. Niacin Inhibited oxLDL-Stimulated Apoptosis and Inflammation in HUVECs

#### 3.2.1. Niacin Attenuated oxLDL-Stimulated Apoptosis of HUVECs

**Figure 4:**
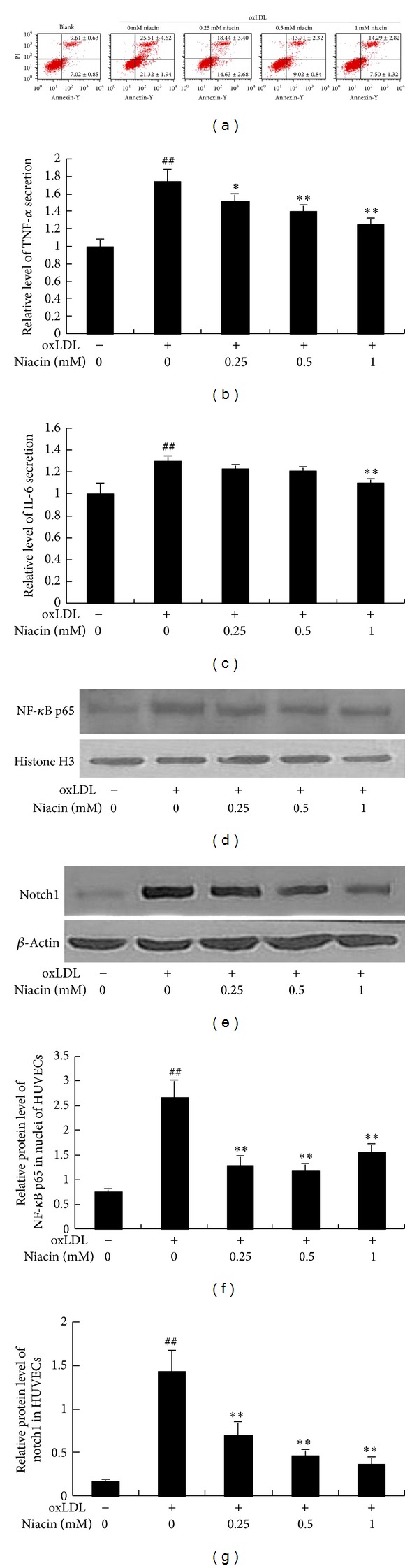
Niacin inhibited oxLDL-stimulated apoptosis and inflammation in HUVECs. (a) shows the ratio of apoptotic HUVECs stained with annexin V-FITC and PI. Representative data of flow cytometry analysis are presented. (b) and (c) show the relative levels of TNF-*α* and IL-6 in the medium of HUVECs. The concentrations of IL-6 and TNF-*α* were determined by ELISA kit. (d) and (e) show the representative images of NF-*κ*B p65 and notch1 protein expression in HUVECs by western blot. (f) and (g) show the IOD ratios of NF-*κ*B p65 and notch1 expression, respectively. Data are presented as mean ± SD. ^##^ *P* < 0.01 versus blank group; *P < 0.05; **P < 0.01 versus oxLDL-treated  group without niacin.

The inflammation and further damage, including apoptosis, of arterial ECs are considered as early and necessary steps of AS as well as thrombosis. In this study, HUVECs apoptosis was analyzed via flow cytometry. After HUVECs were stained with Annexin V-FITC/PI, flow cytometry analysis revealed that apoptosis was induced by 150 *μ*g/mL ox-LDL for 24 h, which was significantly improved by niacin (Figure 4(a)).

#### 3.2.2. Niacin Decreased the Secretion of Inflammatory Cytokines TNF-*α* and IL-6 in oxLDL-Stimulated HUVECs

As shown in Figures 4(b) and 4(c), ox-LDL (150 *μ*g/mL) markedly increased TNF-*α* and IL-6 protein levels by 76% and 31%, respectively, in the medium of HUVECs. Preincubation of cells with niacin (0.25–1 mM) significantly inhibited TNF-*α* expression by 12, 21, and 27%, respectively. Meanwhile, 1 mM niacin lowered IL-6 level by 15% in the medium.

#### 3.2.3. Niacin Suppressed NF-*κ*B p65 and Notch1 Expression in oxLDL-Stimulated HUVECs

Notch signaling pathway plays a key role in the inflammatory progress. It can activate NF-*κ*B and promote inflammatory factors secretion from endothelial cells and macrophages [16]. To further explore the mechanism through which niacin inhibited cell injury, we determined the expressions of nuclear protein NF-*κ*B p65 and notch1 by western blot. The results showed that oxLDL markedly increased the protein levels of active NF-*κ*B p65 and notch1 in HUVECs, which were suppressed by preincubation of cells with niacin in a dose-dependent manner (Figures 4(d), 4(e), 4(f), and 4(g)).

### 3.3. Niacin Suppressed Inflammatory Response Stimulated by oxLDL in THP-1 Macrophages

#### 3.3.1. Niacin Decreased TNF-*α* and IL-6 Protein Secretion in the Medium of THP-1 Macrophages

**Figure 5:**
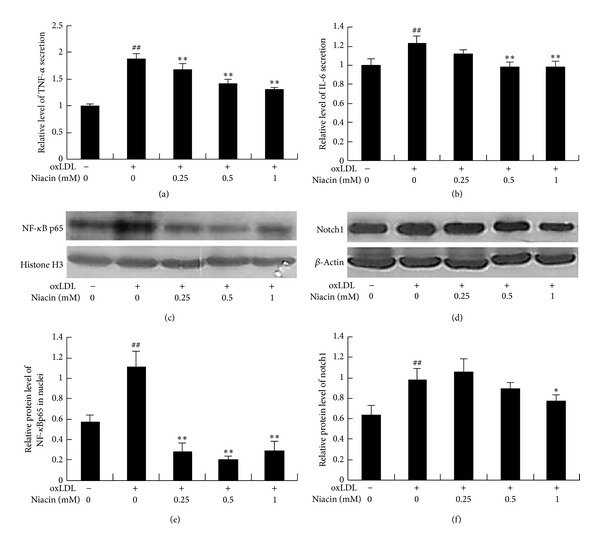
Niacin decreased the secretion of TNF-*α* and IL-6 and inhibited NF-*κ*B and notch1 expression in oxLDL-stimulated THP-1 macrophages. (a) and (b) show the relative levels of TNF-*α* and IL-6 secretion in the medium of THP-1 macrophages. The concentrations of IL-6 and TNF-*α* were determined by ELISA kit. (c) and (d) show the representative images of NF-*κ*B p65 and notch1 protein expression in THP-1 macrophages by western blot. (e) and (f) show the IOD ratios of NF-*κ*B p65 and notch1 expression, respectively. Data are presented as mean ± SD. ^##^ *P* < 0.01 versus blank group; *P < 0.05; **P < 0.01 versus oxLDL-treated group without niacin.

Next, we assessed anti-inflammatory property of niacin in THP-1 macrophages. As shown in Figures 5(a) and 5(b), ox-LDL significantly promoted TNF-*α* and IL-6 secretion by 89% and 23%, respectively, in THP-1 macrophages. Niacin (0.25–1 mM) remarkably inhibited TNF-*α* expression by 11–30% and IL-6 expression by 8–22% in the medium.

#### 3.3.2. Niacin Inhibited NF-*κ*B p65 and Notch1 Protein Expression in oxLDL-Induced THP-1 Macrophages

The effect of niacin on the protein expressions of NF-*κ*B p65 in nuclei and notch1 stimulated by ox-LDL were examined. Results showed that niacin (0.25–1 mM) significantly decreased NF-*κ*B level by 75–83% and niacin (1 mM) decreased notch1 level by 20% (Figures 5(c), 5(d), 5(e), and 5(f)).

### 3.4. Niacin Significantly Lessened Lipid Deposition in the Arterial Wall and Modified Lipoprotein Profile in Plasma via Modulating Cholesterol Metabolism in Liver of Guinea Pigs Fed High Fat Diet

#### 3.4.1. Niacin Significantly Lessened Lipid Deposition in the Arterial Wall of Guinea Pigs Fed High Fat Diet

**Figure 6:**
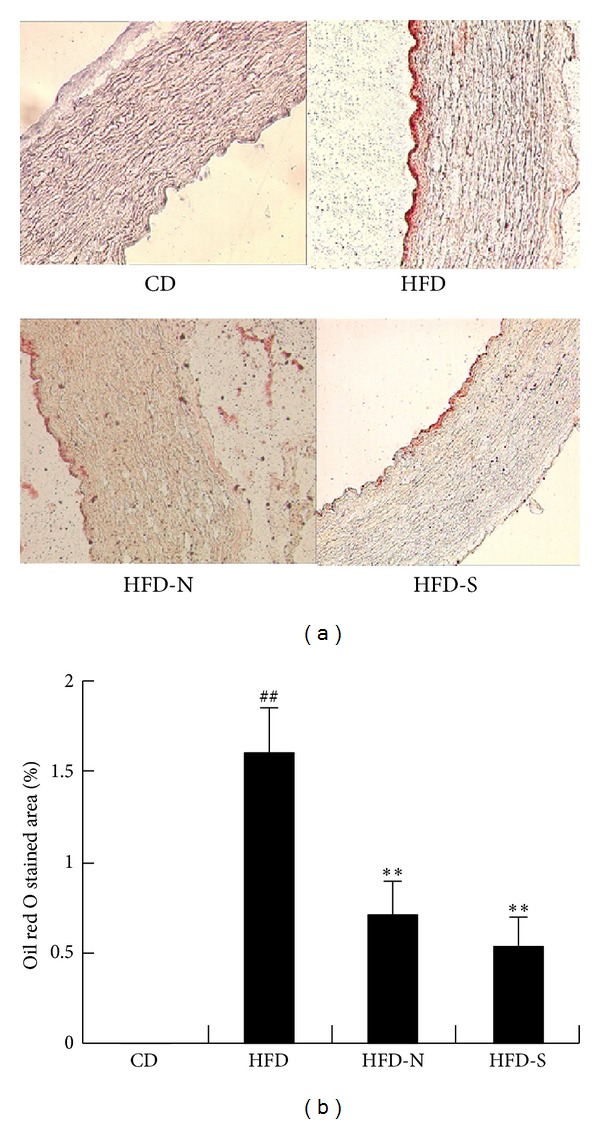
Niacin and simvastatin significantly lessened lipid deposition in the arterial wall of guinea pigs fed high fat diet. Lipid deposition in the aorta wall was analyzed by oil red O staining after treatment for 8 weeks. The quantification of stained lipids was determined by calculating the percentage of the positive area to the total cross-sectional vessel wall area by Image-Pro Plus software. Data are presented as mean ± SD (*n* = 8). ^##^ *P* < 0.01 versus CD group; **P < 0.01 versus HFD group.

Oil red O staining in the aorta was found in HFD group but not in CD group because of chow diet without high fat. Compared with that of HFD group, niacin and simvastatin significantly decreased the percentages of stained area to the total cross-sectional vessel wall by 56% and 67%, respectively (Figure 6). The effect of simvastatin was superior to that of niacin.

#### 3.4.2. Niacin Increased HDL-C and ApoA I Levels and Decreased TG and Non-HDL-C Levels in Plasma of Guinea Pigs Fed High Fat Diet

**Figure 7:**
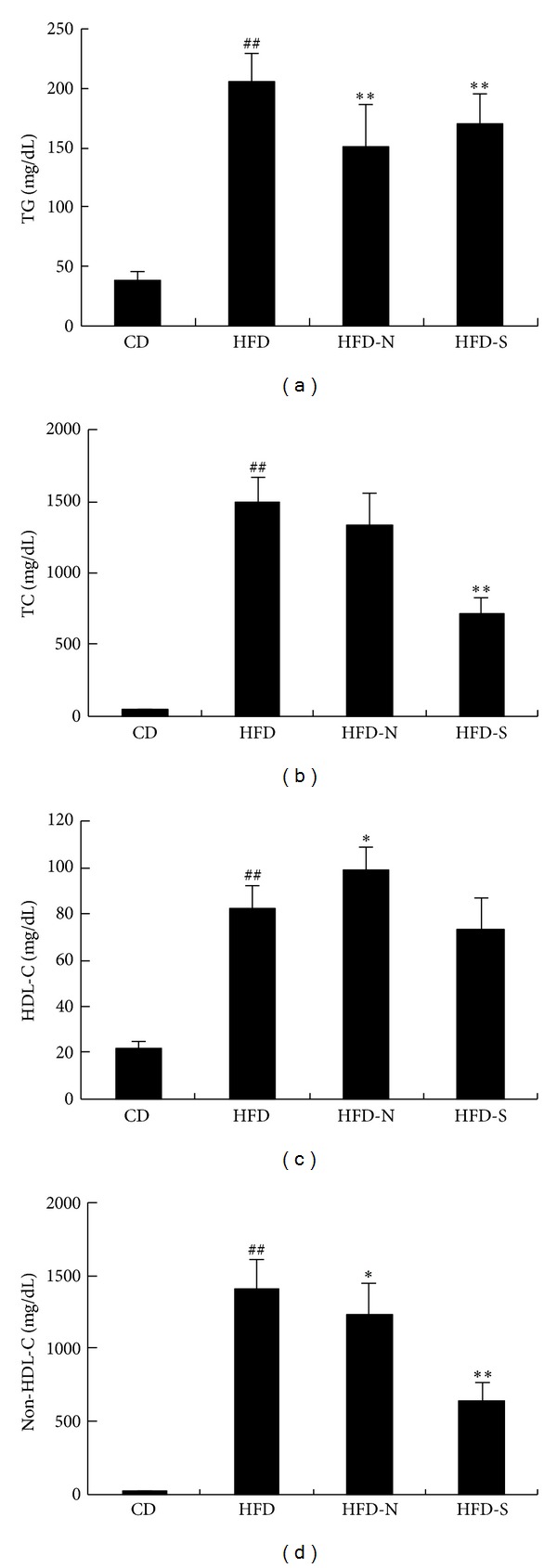
Effect of niacin and simvastatin on plasma lipid of guinea pigs fed high fat diet. The levels of TG (a), TC (b), HDL-C (c), and non-HDL-C (d) in plasma of guinea pigs were determined by enzyme method after 8 weeks' treatment. Data are presented as mean ± SD (*n* = 8). ^##^ *P* < 0.01 versus CD group; *P < 0.05; **P < 0.01 versus HFD group.

As shown in Figure 7, after high fat diet for 8 weeks, the levels of plasma TC, TG, HDL-C, and non-HDL-C were significantly increased in HFD group compared with CD group (*P* < 0.01), which indicated a successful hyperlipidemic model in guinea pigs. Compared with HFD group, niacin decreased the levels of TG and non-HDL-C by 27% and 12%, respectively, and increased HDL level by 21%. Niacin had no statistical influence on TC level in plasma. Compared with HFD group, simvastatin decreased the levels of TG, non-HDL-C, and TC by 18%, 53%, and 51%, respectively. Simvastatin had no significant influence on HDL-C level.

**Figure 8:**
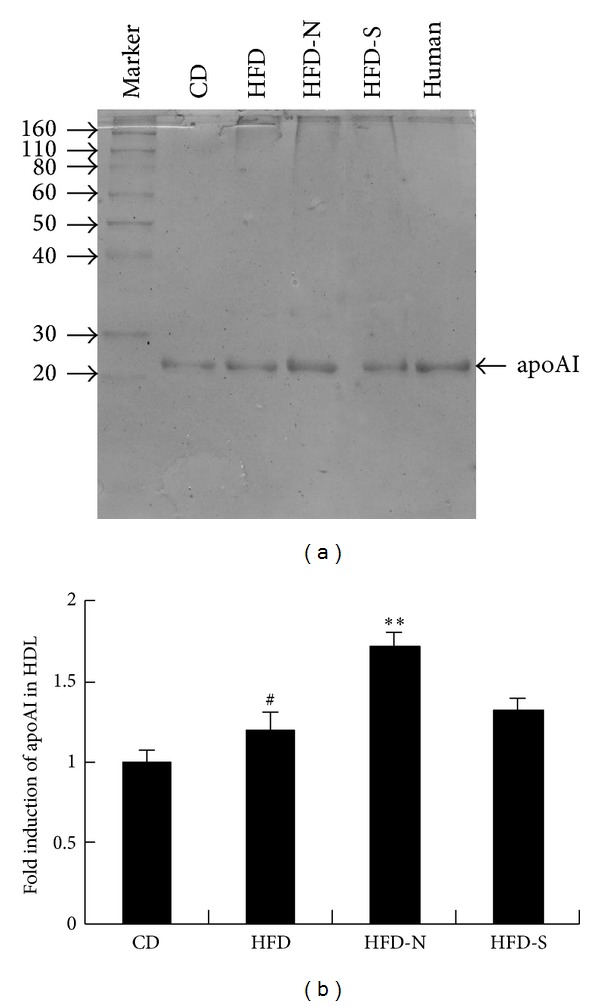
Niacin upregulated apoA I level in plasma of guinea pigs fed high fat diet. HDL (density = 1.09–1.24 g/mL) was separated from plasma of guinea pigs by sequential ultracentrifugation. The SDS-PAGE was performed on 15% SDS polyacrylamide gel, and the apolipoproteins were stained with coomassie brilliant blue. Densitometric quantitation of SDS-PAGE image was analyzed by Image-Pro Plus software. (a) shows the representative SDS-PAGE image of HDL. (b) shows the relative level of apoA I in plasma by densitometric quantitation. Data are presented as mean ± SD of at least three independent experiments. ^#^ *P* < 0.05 versus CD group; **P < 0.01 versus HFD group.

The level of apoA I in plasma was also detected by SDS-PAGE in this study. Compared with that of HFD group, niacin significantly promoted the level of apoA I by 42%, whereas simvastatin had no significant influence on apoA I (Figure 8).

#### 3.4.3. Niacin Significantly Upregulated the mRNA Amount of CYP7A1 in Liver

**Figure 9:**
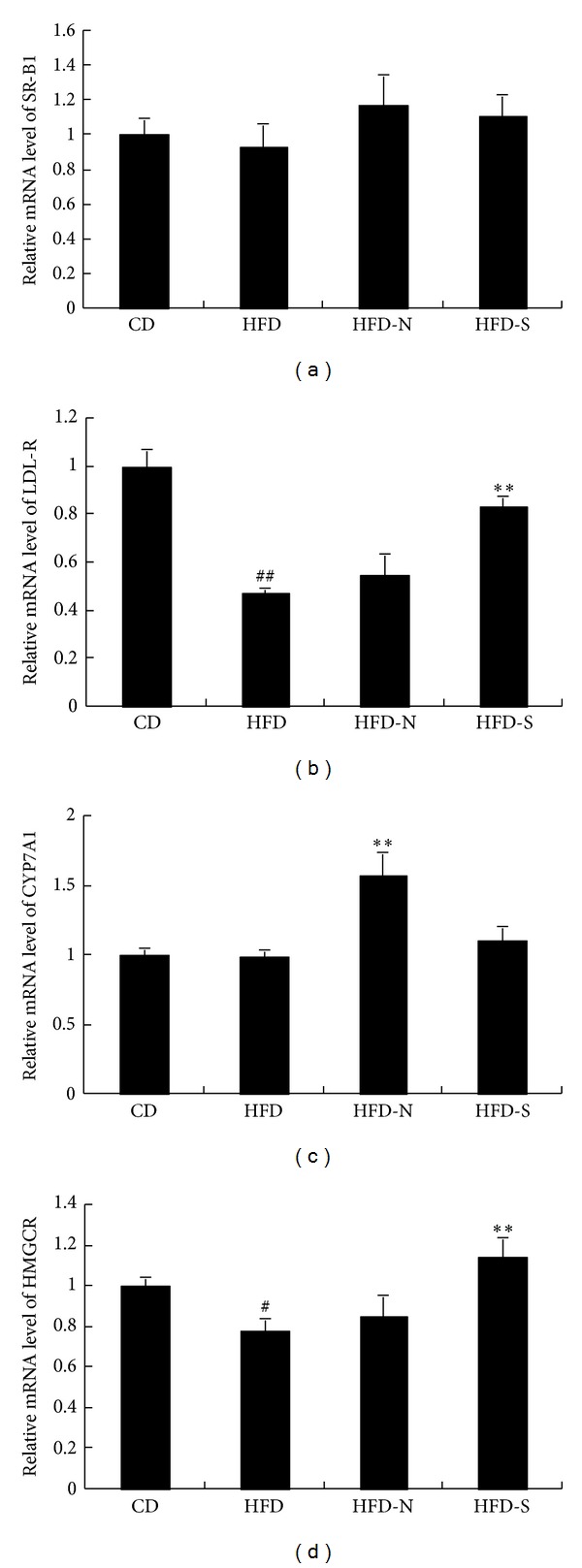
Effect of niacin and simvastatin on the mRNA abundance of SR-B1, LDL-R, CYP7A1, and HMGCR in liver of guinea pigs fed high fat diet. The mRNA levels, which were analyzed by quantitative real-time PCR, were calculated after being adjusted for *β*-actin using the 2^−ΔΔCt^ method. Data are presented as mean ± SD of at least three independent experiments. ^#^ *P* < 0.05; ^##^ *P* < 0.01 versus CD group; **P < 0.01 versus HFD group.

Cholesterol metabolism in liver is a complicated homeostasis involving multiple steps, including cholesterol ingression, synthesis, and conversion. SR-B1 and LDL-R in liver play a critical role in cholesterol ingression. SR-B1 is the HDL receptor on the hepatocyte surface. LDL-R can bind to LDL and VLDL and internalize them into hepatocytes [17]. In the study, we determined the effect of niacin on the expressions of SR-B1 and LDL-R mRNA in liver. As shown in Figures 9(a) and 9(b), after treatment with high fat diet for 8 weeks, the LDL-R mRNA level was downregulated (*P* < 0.01) and the SR-B1 mRNA level was not significantly changed in HFD group. Compared with HFD group, niacin had no significant effect on SR-B1 and LDL-R mRNA levels, but simvastatin upregulated LDL-R mRNA level by 71%.

Cholesterol in liver can be converted into bile acid through cytochrome P450-meidiated oxidation. The rate-limiting enzyme for the dominant pathway of bile acid synthesis is cytochrome P450 7A1 (CYP7A1). As shown in Figure 9(c), compared with HFD group, niacin significantly upregulated the CYP7A1 mRNA level by 59%, whereas simvastatin had no significant influence on its level. HMGCR is the rate-limiting enzyme in the process of cholesterol synthesis. Compared with that of CD group, the mRNA level of HMGCR was significantly decreased in HFD group (*P* < 0.01). Compared with HFD group, simvastatin upregulated the HMGCR mRNA levels by 46%, whereas niacin had no significant influence on its level (Figure 9(d)).

## 4. Discussion

For the first time, to our knowledge, this report demonstrates niacin inhibited vascular inflammation in guinea pig fed high fat diet and suppressed oxLDL-stimulated inflammatory response, even injury, of endothelial cells and macrophages* in vitro*. The result indicates a new mechanism for niacin's protective action on cardiovascular disease in addition to its established effects on lipid metabolism.

The augmentation of inflammatory response has been clearly documented in pathogenesis of vascular impairment. The chronic inflammatory pathogenesis in the arterial wall is as follows. Harmful substances in blood, such as hypercholesterolemia, can induce endothelial dysfunction. This causes the production of ROS and the secretion of cellular adhesion molecules (CAMs), cytokines, and chemokines which facilitate adherence and endothelial transmigration of leukocytes (monocytes and T helper lymphocytes). Monocytes in the arterial wall will be activated by proinflammatory cytokines and differentiated into macrophages. Activated macrophages increase the expression of CAMs and cytokines, which results in recruitment of more leukocytes into the arterial wall, activates the complement pathways of immune system and the acute phase response, stimulates proliferation and migration of smooth muscle cells (SMCs), and promotes fibrous tissue deposition [18]. In progress, the signaling molecule NF-*κ*B is a proinflammatory major switch that can upregulate the expression of lots of cytokines [19]. Activated NF-*κ*B can result in the enhanced efflux of TNF-*α* and IL-6 in serum [20]. Early events in AS are usually driven by NF-*κ*B and the disruption of NF-*κ*B signaling pathway has been shown to slow down the vascular impairment [21].

In the present study we demonstrate that niacin attenuated vascular inflammation induced by high fat diet* in vivo*. The involved evidences are as follows. (1) Niacin lowered the number of macrophages (CD68 positive cells) in the arterial wall and significantly downregulated the inflammatory factors (IL-6 and TNF-*α*) levels in plasma of guinea pigs fed high fat diet. (2) Both immunohistochemistry and western blot analysis indicated niacin suppressed the expression of active NF-*κ*B p65 in nuclei of the arterial wall. The activated NF-*κ*B is reported to form a heterodimer, which usually consists of two proteins, p65 and p50 subunits. The p65 subunit has been demonstrated to exert critical activity on the transcription of many inflammatory genes, including adhesion molecules, cytokines, and chemokines [22]. (3) CRP, an early acute phase reactant, is closely relevant to inflammation. Baseline level of CRP is a strong independent predictor of the risk of future myocardial infarction, peripheral vascular disease, stroke, and vascular death among healthy individuals without known vascular disease [23]. Kuvin et al. have shown that niacin decreased CRP level by 15% in patients with stable coronary artery disease [5]. In patients with metabolic syndrome, after treatment with extended-release niacin (1 g/day) for 52 weeks, their endothelial function was improved by 22% and there was a decrease in CRP level by 20% [24]. Our results also showed niacin slightly lowered CRP level but had no statistical difference (Table 1). (4) Oxidative stress was suppressed by niacin. Oxidative stress is closely related to the inflammation in the arterial wall. Increased ROS production can initiate a cascade of signal transduction, which results in endothelial dysfunction, changes in vascular tone, vascular remodeling, and vascular inflammatory responses [25].

To further confirm the direct anti-inflammatory property of niacin, its effect on oxLDL-induced inflammatory response of endothelial cells and macrophages was studied. oxLDL is pivotal in the development of AS and represents a crucial proinflammatory stimulus [26]. Upon entering into the intima of arteries, oxLDL activates endothelial cells and upregulates adhesion molecule expression and inflammatory factors secretion, all of which contribute to the recruitment of circulating leukocytes. Monocytes and/or macrophages infiltrating the arterial wall take up oxLDL and form “foam cells,” which in turn promote further secretion of inflammatory mediators [27]. Our data indicated niacin remarkably downregulated the secretion of TNF-*α* and IL-6 stimulated by oxLDL in HUVECs and THP-1 macrophages. Notch1 signal pathway is an evolutionarily highly conserved mechanism for communication, which can improve NF-*κ*B activity and have a positive correlation with the development of inflammation [28]. A previous study demonstrates that notch knockout in macrophage markedly downregulates the expression of proinflammatory factors. Instead, when the notch signal pathway is activated, NF-*κ*B expression increases and inflammatory factors secretion is also promoted [29]. Our findings indicated niacin decreased the expressions of NF-*κ*B and notch1 in oxLDL-induced inflammation. Vascular endothelial apoptosis plays an important role in the initiation of AS. It may compromise vessel wall permeability to cytokines, growth factors, lipids, and immune cells and increase coagulation [30]. In this study, we indicated niacin attenuated oxLDL-induced apoptosis of HUVECs.

Taken together, these observations lend initial but compelling evidence that niacin has protective effects on cardiovascular disease through its vascular anti-inflammatory properties in addition to its established effects on lipid metabolism.

Reverse cholesterol transport (RCT) is a potent defense mechanism against AS. It involves transporting cholesterol from peripheral tissues and cells to liver, transforming cholesterol into bile acid, and finally eliminating it from the body [31]. HDL and apoA play a key role in RCT. They can drive cholesterol efflux from macrophage foam cells that reside in vessel intima [32, 33]. Similar to previous studies, our findings also indicated that niacin increased the levels of HDL and apoA I in plasma significantly. In the process of RCT, CYP7A1 is an important regulatory factor which can convert cholesterol to bile acid in liver and be further excreted into intestine for elimination from body with feces. Here, we showed that the mRNA abundance of CYP7A1 in liver was significantly upregulated by niacin treatment. These findings suggest that niacin may modulate plasma lipid and exert antiatherosclerotic effects by promoting RCT progress.

In clinical practice, the oral dose of niacin for lipid-lowering purpose is usually 1–3 g per day, equivalently 14–43 mg/kg per day. According to the formula of Meeh-Rubner equation, we calculate the dose for guinea pig to be about 80–250 mg/kg per day. In addition, a recent report showed that up to 400 mg/kg per day of niacin treatment for 7 days and 100 mg/kg per day of niacin treatment for 4 weeks are safe and do not cause toxicity in rodent models [34]. In this study, the applied dose of niacin for guinea pig is 100 mg/kg/d, which has pharmacologic effects and does not have various toxicities based on the changes in food intake, body weight, and behaviors. In human, plasma level of niacin was found to be about 0.3 mM after oral ingestion of 1 g niacin [35, 36]. Since the reported plasma concentrations are one time measurement, it is unclear whether these levels reflect trough or peak values. Furthermore, there is no reported clinical data available regarding plasma concentration of niacin after ingestion of 3 g niacin. Extrapolation of the available data using oral dose of 1 g niacin would indicate that the 3 g niacin oral administration may lead to the plasma concentrations of niacin in the range of 0.8–1 mM. In our* in-vitro* studies we used 0.25 to 1 mM niacin.

Statins are the first-line therapy in the treatment of cholesterol-induced atherosclerotic cardiovascular diseases [37]. They suppress the conversion of HMG-CoA to mevalonate by competitively blocking HMGCR; therefore, the endogenous cholesterol synthesis in the liver is remarkably reduced. Consequently, it results in the increase of LDL-R expression on hepatic cell surfaces and the decrease of LDL-C in plasma [38]. Meanwhile, inhibition of cholesterol synthesis causes an overexpression of HMGCR due to a feedback-regulatory mechanism [39]. Similar to previous studies, our findings also revealed that simvastatin significantly decreased the level of non-HDL-C in plasma followed by upregulating LDL-R expression in liver and the HMGCR mRNA level in liver was increased in a compensatory way.

## 5. Conclusion

In summary, our data presented herein support the novel concept that niacin has vascular anti-inflammatory and potentially vascular-protective property which is independent of its effect on lipid regulation. The anti-inflammatory property of niacin is realized by downregulating nuclear transcription factor-*κ*B signaling pathway. Meanwhile, our finding also suggests that niacin modulates plasma lipid by stimulating the expression of factors involved in the process of RCT and promoting RCT.

## Figures and Tables

**Table 1:** Effect of niacin and simvastatin on plasma inflammatory markers (CRP, IL-6, and TNF-*α*) of guinea pig fed high fat diet.

Group	CRP (ng/L)	IL-6 (ng/L)	TNF-*α* (ng/L)
CD	3169.9 ± 219.7	258.8 ± 25.2	130.8 ± 9.2
HFD	3211.3 ± 153.8	265.8 ± 24.2	143.0 ± 15.7
HFD-N	3023.1 ± 180.6	215.2 ± 38.5**	117.9 ± 17.9**
HFD-S	2955.7 ± 257.8*	236.7 ± 21.6*	114.4 ± 19.3**

The contents of CRP, IL-6, and TNF-*α* in guinea pig plasma were measured by ELISA method after 8 weeks' treatment. Data are presented as mean ± SD (*n* = 8). *P < 0.05; **P < 0.01 versus HFD group.
